# Microglia change from a reactive to an age-like phenotype with the time in culture

**DOI:** 10.3389/fncel.2014.00152

**Published:** 2014-06-02

**Authors:** Cláudia Caldeira, Ana F. Oliveira, Carolina Cunha, Ana R. Vaz, Ana S. Falcão, Adelaide Fernandes, Dora Brites

**Affiliations:** ^1^Research Institute for Medicines – iMed.ULisboa, Faculdade de Farmácia, Universidade de LisboaLisboa, Portugal; ^2^Centro de Investigação Interdisciplinar Egas Moniz, Egas Moniz – Cooperativa de Ensino Superior, CRL, Campus UniversitárioMonte de Caparica, Portugal; ^3^Department of Biochemistry and Human Biology, Faculdade de Farmácia, Universidade de LisboaLisboa, Portugal

**Keywords:** autophagic capacity, *in vitro* cell aging, microglia, microRNAs, migration, phagocytosis, reactivity, senescence

## Abstract

Age-related neurodegenerative diseases have been associated with chronic neuroinflammation and microglia activation. However, cumulative evidence supports that inflammation only occurs at an early stage once microglia change the endogenous characteristics with aging and switch to irresponsive/senescent and dystrophic phenotypes with disease progression. Thus, it will be important to have the means to assess the role of reactive and aged microglia when studying advanced brain neurodegeneration processes and age-associated related disorders. Yet, most studies are done with microglia from neonates since there are no adequate means to isolate degenerating microglia for experimentation. Indeed, only a few studies report microglia isolation from aged animals, using either short-term cultures or high concentrations of mitogens in the medium, which trigger microglia reactivity. The purpose of this study was to develop an experimental process to naturally age microglia after isolation from neonatal mice and to characterize the cultured cells at 2 days *in vitro* (DIV), 10 DIV, and 16 DIV. We found that 2 DIV (young) microglia had predominant amoeboid morphology and markers of stressed/reactive phenotype. In contrast, 16 DIV (aged) microglia evidenced ramified morphology and increased matrix metalloproteinase (MMP)-2 activation, as well as reduced MMP-9, glutamate release and nuclear factor kappa-B activation, in parallel with decreased expression of Toll-like receptor (TLR)-2 and TLR-4, capacity to migrate and phagocytose. These findings together with the reduced expression of microRNA (miR)-124, and miR-155, decreased autophagy, enhanced senescence associated beta-galactosidase activity and elevated miR-146a expression, are suggestive that 16 DIV cells mainly correspond to irresponsive/senescent microglia. Data indicate that the model represent an opportunity to understand and control microglial aging, as well as to explore strategies to recover microglia surveillance function.

## INTRODUCTION

Microglia are the first line of defense against brain injury. In the healthy brain, microglia actively survey surrounding parenchyma via dynamic movement of processes (Nimmerjahn et al., 2005) and are kept in a relatively quiescent state, in part due to specific signals derived from neurons and astrocytes (Cardona et al., 2006; Lyons et al., 2007). Upon brain injury or changes of central nervous system (CNS) homeostasis, microglia are capable of acquiring diverse and complex phenotypes, allowing them to participate in the cytotoxic response, immune regulation, and injury resolution. The classical pro-inflammatory M1 phenotype is cytotoxic and release pro-inflammatory cytokines while the M2 polarization contributes to neuroprotection through the release of anti-inflammatory cytokines and growth factors (Chhor et al., 2013; Evans et al., 2013). These transitional phenotypes may exert beneficial or destructive effects depending on the stimuli, their duration and the environment they encounter (Schwartz et al., 2006). Thus, balance between M1 and M2 phenotypes can be considered a desirable therapeutic goal.

Age-related CNS disorders have been related with chronic and progressive neuronal loss but also with chronic mild neuroinflammation involving activated/primed microglia (Maezawa et al., 2011; Williamson et al., 2011). These cells showed to switch from M2 to M1 phenotype with age and disease progression (Solito and Sastre, 2012; Varnum and Ikezu, 2012). However, other studies claim that neuroinflammation is only present in the early stages of Ahlzheimer’s disease (AD), once lately disappears (Wojtera et al., 2012) and that, instead, microglia become senescent/dystrophic (Graeber and Streit, 2010) and less responsive to stimulation with age (Njie et al., 2012; Streit and Xue, 2012). The dysmorphic characteristics of aged microglia suggested that, rather than maintaining an overactivated state, microglia may display decreased ability to mount a normal response to injury. Indeed, reduced migration (Damani et al., 2011), clearance (Li, 2013) and production of neurotrophic factors (Ma et al., 2013), as well as inability to shift from a pro-inflammatory to an anti-inflammatory state to regulate injury and repair have been observed in aged microglia (Norden and Godbout, 2013) and related with senescence (Streit and Xue, 2012). These changes in microglia potentially contribute to an increased susceptibility and neurodegeneration as a function of age. Accordingly, non-steroidal anti-inflammatory drugs (NSAIDs) were only successful when administered before the development of neurodegeneration (Weggen et al., 2001). If administered in later stages of disease they showed to be detrimental (Martin et al., 2008), reinforcing that microglia may switch from a reactive to an irresponsive phenotype along the progression of AD and other age-associated CNS disabilities. Restraining of aged microglia may weak even more the already decreased neuroprotective properties of the cell in removing extracellular protein aggregates. These changes in microglia neuroprotective properties will potentially contribute to enhance neurodegeneration and susceptibilities with aging and reveal the need of adequate experimental models to follow the changes in microglia performance accordingly to cell senescence.

Most of the work intended to evaluate the neurodegenerative network associated with aging has used cultures of microglia derived from early postnatal brains, which differ from adult or aged ones (Harry, 2013). Recently, a few studies compared behavior of microglia isolated from animals at different ages. In these studies young and aged microglia were isolated using a Percol-based method (von Bernhardi et al., 2011; Njie et al., 2012) or distinctly isolated using a mild-trypsinization method for embryonic/neonatal microglia and Percol-based method for adult and aged microglia (Lai et al., 2013). In addition, these cells were analyzed either 24–48 h after isolation (Njie et al., 2012; Lai et al., 2013) or following trypsinization when kept in culture for several weeks in the presence of conditioned medium containing increased levels of mitogens (von Bernhardi et al., 2011). Such methods may promote microglia activation and bias the translation of culture findings, since it has been suggested that microglia may need some time in culture to recover its quiescent state (Cristóvão et al., 2010). Moreover, there are no means to isolate degenerating microglia for experimentation (Njie et al., 2012) once only the more resistant ones will survive to the isolation procedure. Nevertheless, the hypothesis of microglia senescence during aging and related neurodegenerative diseases emerged as a key determinant (Luo et al., 2010). *In vitro* aging of astrocytes and neurons has demonstrated to be associated with different cell response to stimuli, with the younger cells evidencing an increased reactivity when compared to the older ones (Falcão et al., 2005, 2006). In addition, it was shown that the repeated stimulation of the microglia cell line BV2 with lipopolysaccharide (LPS) lead to cell senescence corroborating the idea that sustained neuroinflammation may ultimately contribute to a microglia senescent phenotype (Yu et al., 2012). Therefore, we decided to isolate microglia from neonatal mice and culture cells from 2 days *in vitro* (DIV) until 16 DIV, similarly to what we previously did with neurons and astrocytes, and to explore aging-related differences in functional response characteristics associated to “young” and “aged” microglia phenotypes. We assessed changes in microglia morphology, nuclear factor kappa-B (NF-κB) signaling pathway activation, Toll-like receptor (TLR) expression, phagocytic ability and migration capacity, as well as cell death, inflammatory microRNA (miRNA) profiling, autophagy and senescence-associated β-galactosidase (SA-β-gal) in mice primary cortical cell cultures maintained up to 16 DIV.

## MATERIALS AND METHODS

### ANIMALS

Animal care followed the recommendations of the European Convention for the Protection of Vertebrate Animals Used for Experimental and Other Scientific Purposes (Council Directive 86/609/EEC) and National Law 1005/92 (rules for protection of experimental animals). All animal procedures were approved by the Institutional animal care and use committee. Every effort was made to minimize the number of animals used and their suffering.

### PRIMARY CULTURE OF MICROGLIA

Mixed glial cultures were prepared from 1-to-2 day-old CD1 mice as previously described (McCarthy and de Vellis, 1980), with minor modifications (Gordo et al., 2006). Cells (4 × 10^5^ cells/cm^2^) were plated on uncoated 12-well tissue culture plates (with 18 mm coverslips) or 75-cm^2^ culture flasks in culture medium [DMEM-Ham’s F-12 medium supplemented with 2 mM L-glutamine, 1 mM sodium pyruvate, non-essential amino acids 1×, 10% fetal bovine serum (FBS), and 1% antibiotic-antimycotic solution] and maintained at 37°C in a humidified atmosphere of 5% CO_2_.

Microglia were isolated as previously described by Saura et al. (2003). Briefly, after 21 days in mixed culture, microglia were obtained by mild trypsinization with a trypsin-EDTA solution diluted 1:3 in DMEM-Ham’s F12 for 45–60 min. The trypsinization resulted in detachment of an upper layer of cells containing all the astrocytes, whereas the microglia remained attached to the bottom of the well. The medium containing detached cells was removed and the initial mixed glial-conditioned medium was added. 

Mixed cultures were maintained in culture for 21 days to achieve the maximal yield and purity of the cultures. In fact, contamination by astrocyte and neurons was less than 2 and 0%, respectively, as assessed by immunocytochemical staining with a primary antibody against GFAP and MAP-2, respectively, followed by a species-specific fluorescent-labeled secondary antibody (Silva et al., 2010).

### CHARACTERIZATION OF MICROGLIA ALONG THE DAYS IN CULTURE

After mild trypsinization, attached cells on uncoated 18-mm coverslips were maintained in culture until reaching 2, 10, or 16 DIV for characterization, with medium replaced every 4 days. Microglia characterization was first performed considering cell morphology and NF-κB activation, at these three time-points, and thereafter only at 2 and 16 DIV for additional properties related with migration ability, phagocytic capacity, differential cell reactive ability and markers of cell senescence.

### CELL MORPHOLOGICAL ANALYSIS

For morphological analysis, cells were fixed for 20 min with freshly prepared 4% (w/v) paraformaldehyde in phosphate-buffer saline (PBS) and a standard immunocytochemical technique was performed using a primary antibody raised against Iba-1 (rabbit, 1:250; Wako Pure Chemical Industries Ltd, Osaka, Japan), and a secondary Alexa Fluor 594 goat anti-rabbit (1:1000; Invitrogen Corporation, Carlsbad, CA, USA). To identify the total number of cells, microglial nuclei were stained with Hoechst 33258 dye. Fluorescence was visualized using an AxioCam HRm camera adapted to an AxioSkope^®^ microscope (Zeiss). Pairs of U.V. and red-fluorescence images of ten random microscopic fields (original magnification: 400×) were acquired per sample. To quantitatively characterize microglia morphology we used the particle measurement feature in ImageJ (1.47v, USA) to automatically measure the 2D area, perimeter, and Feret’s diameter of single microglia cells. Feret’s (maximum) diameter, a measure of cell length, is the greatest distance between any two points along the cell perimeter. We also evaluated the transformation index, first defined by Fujita et al. (1996) as [perimeter of cell (μm)]^2^/4π [cell area (μm^2^)], which categorizes microglia ramification status. A cell with long processes and a small soma exhibits a large index that is dependent on cell shape but independent of cell size.

### DETECTION OF NF-κB ACTIVATION

For immunofluorescence detection of NF-κB nuclear translocation, cells were fixed as above and a standard indirect immunocytochemical technique was carried out using a polyclonal rabbit anti-p65 NF-κB subunit antibody (1:200; Santa Cruz Biotechnology^®^, CA, USA) as the primary antibody, and an anti-rabbit Cy2 as the secondary antibody (1:1000; GE Healthcare, Chalfont St. Giles, UK). Microglial nuclei were stained with Hoechst 33258 dye. Fluorescence was visualized and acquired as above. NF-κB positive nuclei were identified by localization of the NF-κB p65 subunit staining exclusively at the nucleus and total cells were counted to determine the percentage of NF-κB-positive nuclei at each cell DIV group.

### DETERMINATION OF CELL DEATH

We used phycoerythrin-conjugated annexin V (annexin V-PE) and 7-amino-actinomycin D (7-AAD; Guava Nexin^®^ Reagent, #4500-0450, Millipore) to determine the percentage of viable, early-apoptotic and late-apoptotic/necrotic cells by flow cytometry. After incubation adherent microglia were collected by trypsinization and added to the cells present in the incubation media. After centrifugation cells were resuspended in PBS containing 1% bovine serum albumin, stained with annexin V-PE and 7-AAD, following manufacturer’s instructions, and analyzed on a Guava easyCyte 5HT flow cytometer (Guava Nexin^®^ Software module, Millipore), as previously described (Barateiro et al., 2012). Three populations of cells can be distinguished by this assay: viable cells (annexin V-PE and 7-AAD negative), early apoptotic cells (annexin V-PE positive and 7-AAD negative), and late stages of apoptosis or dead cells (annexin V-PE and 7-AAD positive).

### ASSESSMENT OF MICROGLIA MIGRATION

Cell migration assays were performed in a 48-well microchemotaxis Boyden chamber (Neuro Probe, Gaithersburg, MD, USA), as previously described (Miller and Stella, 2009), with minor modifications. The bottom wells, filled with ATP (10 μM), a known chemoattractant for microglia migration, served as positive controls. The 8 μm diameter polycarbonate membranes with polyvinylpyrrolidone (PVP) surface treatment was equilibrated in control medium and after chamber set up, 50 μl of cell suspension containing 2 × 10^4^ cells was added to each top well. After 6 h incubation in a CO_2_ incubator at 37°C for microglial migration, membrane was fixed with cold methanol and cells stained with 10% Giemsa in PBS. Non-migrated cells on the upper side of the membrane were wiped off with a filter wiper. The rate of migration was determined by counting cells on the lower membrane surface in 10 microscopic fields to cover all the well, acquired using a Leica DFC490 camera adapted to an AxioSkope HBO50 microscope. For each experiment, at least three wells per condition were analyzed.

### EVALUATION OF PHAGOCYTIC PROPERTIES OF MICROGLIA

To evaluate the phagocytic capacity of the primary microglial cultures, cells collected at 2 and 16 DIV were incubated with 0.0025% (w/w) 1 μm fluorescent latex beads (Sigma Chemical Co., St. Louis, MO, USA) for 75 min at 37°C and fixed with freshly prepared 4% (w/v) paraformaldehyde in PBS. Microglia were stained for Iba-1, nuclei counterstained with Hoechst dye, and fluorescence was visualized and acquired as above. The number of ingested beads per cell was counted. Results are presented as mean number of ingested beads per cell and as the percentage of cells that phagocytosed <5, 5–10, or >10 beads.

### DETERMINATION OF SUPPLEMENTARY FEATURES OF MICROGLIA REACTIVE ABILITY

We used several markers to assess microglia reactive ability, such as the concentration of glutamate and the activation of matrix metalloproteinase (MMP)-2 and MMP-9 in the extracellular media, together with the expression of TLR-2, TLR-4, miR-124 and miR-155.

Glutamate content in the media derived from microglial cultures was determined as described before (Silva et al., 2012) by an adaptation of the L-glutamic acid kit (Roche), using a 10-fold volume reduction. The reaction was performed in a 96-well microplate and the absorbance measured at 490 nm. A calibration curve was used for each assay. All samples and standards were analyzed in duplicate and the mean value was used.

Detection of MMPs activity was performed as previously mentioned (Silva et al., 2010). Aliquots of culture supernatant were analyzed by SDS-PAGE zymography in 0.1% gelatine-10% acrylamide gels under non-reducing conditions. After electrophoresis, gels were washed for 1 h with 2.5% Triton X-100 (in 50 mM Tris pH7.4; 5 mM CaCl_2_; 1 μM ZnCl_2_) to remove SDS and renature the MMP species in the gel. Then the gels were incubated in the developing buffer (50 mM Tris pH7.4; 5 mM CaCl_2_; 1 μM ZnCl_2_) overnight to induce gelatine lysis. For enzyme activity analysis, the gels were stained with 0.5% Coomassie Brilliant Blue R-250 and destained in 30% ethanol/10% acetic acid/H_2_O. Gelatinase activity, detected as a white band on a blue background, was quantified by computerized image analysis and normalized with total cellular protein.

Determination of TLR-2 and TLR-4 mRNA expression was performed by RealTime PCR as usual in our laboratory (Barateiro et al., 2013). Total RNA was extracted from microglia using TRIzol^®^ (LifeTechnologies), according to manufacturer’s instructions. Total RNA was quantified using Nanodrop ND-100 Spectrophotometer (NanoDrop Technologies, Wilmington, DE, USA). Aliquots of 0.5 μg of total RNA were treated with DNase I and then reverse transcribed into cDNA using oligo-dT primers and SuperScript II Reverse Transcriptase under the recommended conditions. Quantitative RealTime-PCR (qRT-PCR) was performed using β-actin as an endogenous control to normalize the expression level of TLR-2 and TLR-4 transcription factors. The following sequences were used as primers: TLR-2 sense 5′-TGCTTTCCTGCTGAAGATTT-3′ and anti-sense 5′-TGTACCGCAACAGCTTCAGG-3′; TLR-4 sense 5′-ACCTGGCTGGTTTACACGTC-3′ and anti-sense 5′-GTGCCAGAGACATTGCAGAA-3′; β-actin sense 5′-GCTCCGGCATGTGCAA-3′ and anti-sense 5′-AGGATCTTCATGAGGTAGT-3′. qRT-PCR was performed on a 7300 Real time PCR System (Applied Biosystems) using a SYBR Green qPCR Master Mix (Thermo Scientific). The PCR was performed in 96 well plates with each sample performed in triplicate, and no-template control was included for each amplificate. qRT-PCR was performed under optimized conditions: 94°C at 3 min followed by 40 cycles at 94°C for 0.15 min, 62°C for 0.2 min and 72°C for 0.15 min. In order to verify the specificity of the amplification, a melt-curve analysis was performed, immediately after the amplification protocol. Non-specific products of PCR were not found in any case. Relative mRNA concentrations were calculated using the Pfaffl modification of the ΔΔCT equation (CT, cycle number at which fluorescence passes the threshold level of detection), taking into account the efficiencies of individual genes. The results were normalized to β-actin in the same sample and the initial amount of the template of each sample was determined as relative expression by the formula 2-ΔΔCT. ΔCT is a value obtained, for each sample, by the difference between the mean CT value of each gene and the mean CT value of β-actin. ΔΔCT of one sample is the difference between its ΔCT value and ΔCT of the sample chosen as reference, in our case the 2 DIV cells.

Expression of miR-124 and miR-155, which has been related with microglia activation phenotype, was performed by qRT-PCR. Total RNA was extracted from primary microglia cultures using the miRCURY^TM^ Isolation Kit – Cells (Exiqon), according to the manufacturer’s recommendations for cultured cells. Briefly, after cell lysis, the total RNA was adsorbed to a silica matrix, washed with the recommended buffers an eluted with 35 μl RNase-free water by centrifugation. After RNA quantification, cDNA conversion for miRNA quantification was performed with the universal cDNA Synthesis Kit (Exiqon) using 5 ng total RNA according to the following protocol: 60 min at 42°C followed by heat-inactivation of the reverse transcriptase for 5 min at 95°C. qRT-PCR was performed using an Applied Biosystems 7300 Sequence Detection system and 96-well plates. For miRNA quantification the miRCURY LNA^TM^ Universal RT microRNA PCR system (Exiqon) was used in combination with pre-designed primers (Exiqon) for miR-124, miR-155 and SNORD110 (reference gene). The reaction conditions consisted of polymerase activation/denaturation and well-factor determination at 95°C for 10 min, followed by 50 amplification cycles at 95°C for 10 s and 60°C for 1 min (ramp-rate of 1.6°/s). The miRNA fold change with respect to 2 DIV cells was determined by the Pfaffl method, taking into consideration different amplification efficiencies of miRNAs in all experiments. The amplification efficiency for each target was determined according to the formula: *E* = 10^(-1/S)^ – 1, where S is the slope of the obtained standard curve.

### ASSESSMENT OF MICROGLIA SENESCENCE

Microglia senescence was evaluated by determining the activity of SA-β-gal, expression of miR-146a and capacity to undergo autophagy. Microglial SA-β-gal activity was determined using the Cellular senescence assay kit (Millipore), according to the manufacturer instructions. Microglial nuclei were counterstained with hematoxylin. Brightfield microscopy images of 10 random microscopic fields were acquired per sample. The number of turquoise stained microglia (SA-β-gal-positive cells) was counted to determine the percentage of senescent cells.

To confirm the senescent status of microglia it was also assessed the expression of the senescence-related miR-146a by qRT-PCR. Total RNA was extracted and expression of miR-146a was assayed using pre-designed primers (Exiqon) for miR-146a and SNORD110 (reference gene) as described above.

Autophagy was determined by both immunocytochemistry of microtubule-associated-protein-light-chain-3 (LC3) punctate and Western Blot detection of LC3 and Beclin-1 bands. For immunocytochemistry, cells were fixed as above and standard immunocytochemical technique was performed using a primary antibody raised against LC3 protein (rabbit, 1:500; Cell Signaling Technology Inc., MA, USA), and a secondary Alexa Fluor 488 goat anti-rabbit antibody (1:1000; Invitrogen Corporation, CA, USA). To identify the total number of cells, microglial nuclei were stained with Hoechst 33258 dye. Fluorescence was visualized and images acquired as above. The method is based on the increased localization of LC3 autophagosomes when autophagy is induced. Thus, the punctate fluorescence produced by LC3 staining provides a sensitive and specific indicator of autophagy (Aoki et al., 2008). Microglial cells presenting LC3 punctate were counted and the percentage of LC3 punctate-positive cells relatively to total microglia was determined. Detection of LC3-II, which is associated with autophagic vesicles (Kabeya et al., 2000), and Beclin-1 bands was processed by Western Blot as usual in our laboratory (Barateiro et al., 2012). Cells were washed in ice-cold PBS, lysed in a buffer containing 20 mM Tris-HCl (pH 7.5), 150 mM NaCl, 1 mM Na2EDTA, 1 mM ethylene glycoltetraacetic acid, 1% (v/v) Triton X-100, 2.5 mM sodium pyrophosphate, 1 mM β-glycerophosphate, 1 mM Na3VO4, 1 μg/mL leupeptine, and 1 mM phenylmethylsulfonyl fluoride, and sonicated for 20 s. The lysate was centrifuged at 14,000 *g* for 10 min at 4°C and the supernatants were collected and stored at -80°C. Protein concentrations were determined using BioRad protein assay (BioRad). Cell extracts containing equal amounts of protein (50 μg) were separated on sodium dodecyl sulfate-polyacrylamide gel electrophoresis and transferred to a nitrocellulose membrane. The membranes were blocked with 5% non-fat milk, incubated with the primary antibody overnight at 4°C [rabbit anti-LC3B (1:1000; #2775, Cell Signaling), mouse anti-Beclin-1 (1:500; #MABC34, MerckMillipore) or mouse anti-β-actin (1:5,000; Sigma)], and then with a horseradish peroxidase-labeled secondary antibody for 1 h at room temperature. After extensive washes, immunoreactive bands were detected by LumiGLO^®^ (Cell Signaling, Beverly, MA, USA) and visualized by autoradiography with Hyperfilm ECL. Results were normalized to β-actin expression and expressed as fold vs. vehicle-treated cells.

### STATISTICAL ANALYSIS

Significant differences between the parameters evaluated were determined by the two-tailed Student’s *t*-test performed on the basis of equal and unequal variance, as appropriate. Comparison of more than two groups (microglia morphology, NF-κB activation) was done by ANOVA using GraphPad Prism 5 (GraphPad Software Inc., San Diego, CA, USA) followed by multiple comparisons Bonferroni *post hoc* correction. *p* value less than 0.05 were considered statistically significant.

## RESULTS

### *IN VITRO* AGING CHANGES MICROGLIA MORPHOLOGY TO A MORE RAMIFIED CELL SHAPE

**FIGURE 1:**
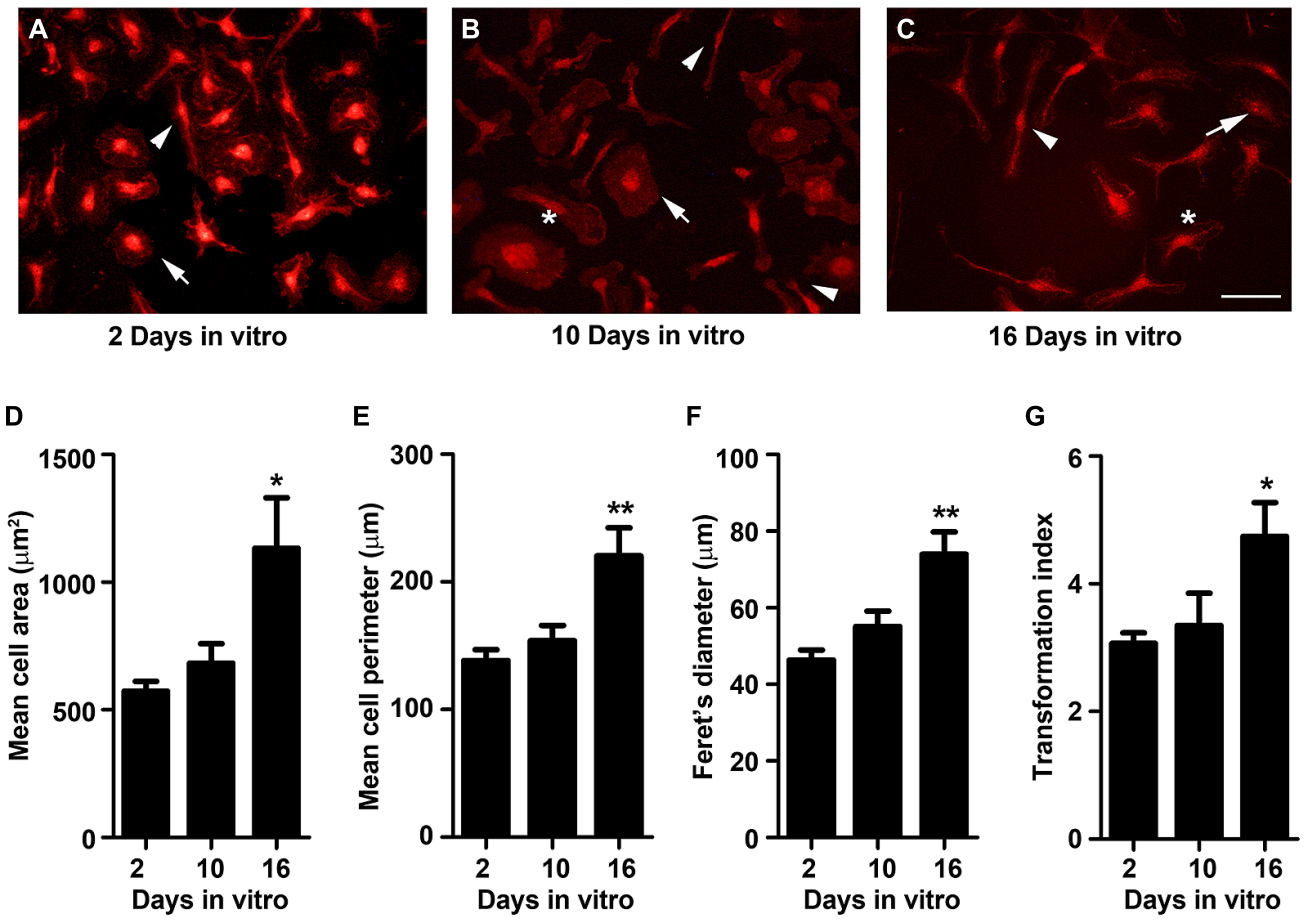
**Microglia morphology changes from amoeboid to a more ramified shape with cell aging in culture.** Microglial cells were kept in culture for 2, 10, and 16 days *in vitro* (DIV), immunostained for Iba-1 and their morphology analyzed. **(A)** At 2 DIV, microglia were amoeboid with ovoid shape (arrow) and only a few showed a ramified bipolar morphology (arrowhead). **(B) **At 10 DIV, microglia became more heterogeneous with more cells presenting a ramified morphology (arrowhead), bearing a single large lamellipodia (*) and some a larger amoeboid shape (arrowhead). **(C)** At 16 DIV cells exhibited distinct polarized populations including ramified rod-like microglia (arrowhead), bipolar microglia with shorter processes (*) and residual amoeboid cells (arrow). Microglia area **(D)**, perimeter **(E)**, and Feret’s diameter **(F)** values were measured using the computer program ImageJ; transformation index values **(G)** were calculated as [perimeter of cell (μm)]^2^/4π [cell area (μm^2^)]. Cultures, *n* = 4 per group. *Post hoc* Bonferroni test, *p < 0.05 and **p < 0.01 vs. 2 DIV cells. Each value represents the mean ± SEM. Scale bar equals 50 μm.

Phenotypic changes in microglia are often accompanied by a morphological transformation, which has been widely used to categorize different activation states. In general, ramified quiescent microglia changes to an activated state displaying larger somata and shorter, coarser cytoplasmic processes progressing to a full amoeboid morphology (Fujita et al., 1996; Kozlowski and Weimer, 2012). Interestingly, microglia isolated from adult and aged animals show a propensity to acquire a more ramified morphology with thicker and more extensive processes (Lai et al., 2013), indicative of a less activated phenotype with age. So, we started by characterizing microglia morphology at 2, 10, and 16 DIV, following immunollabeling with the cell-specific marker Iba-1. As shown in **Figure 1**, diverse morphological forms of microglia may be observed throughout cell culturing. The microglial cells at 2 DIV were almost exclusively amoeboid, most frequently evidencing an ovoid shape with a few cells presenting fusiform shape (**Figure 1A**). At 10 DIV, microglia evidence a more heterogeneous morphology with an increased number of cells showing a ramified morphology, bearing typically one or two large processes or a single large lamellipodia, together with larger amoeboid forms (**Figure 1B**). Microglia cultures at 16 DIV still exhibited distinct polarized populations showing rod-like microglia, bipolar microglia with shorter processes and the residual amoeboid cells (**Figure 1C**). To quantitatively evaluate the effect of age on microglia morphology we measured the area, perimeter, and Feret’s maximum diameter of microglia (**Figures 1D–F**). Consistent with a transformation of amoeboid to microglia ramified forms, the area, perimeter and the Feret’s maximum diameter significantly increased at 16 DIV (~2.0-, ~1.6-, and ~1.6-fold, respectively, *p* < 0.05). Analysis of the transformation index value, a dimensionless number that reflects the degree of process extension, revealed a continuum of microglial phenotypes between the amoeboid and the ramified morphologies (**Figure 1G**). While younger cultures with a predominant amoeboid microglia shape present a low transformation index, older cultures with a more heterogeneous morphological repertoire, involving cells with amoeboid and ramified morphologies, displayed an increased transformation index (~1.6-fold, *p* < 0.05).

### *IN VITRO* AGING REDUCES MICROGLIA NF-κB ACTIVATION

**FIGURE 2:**
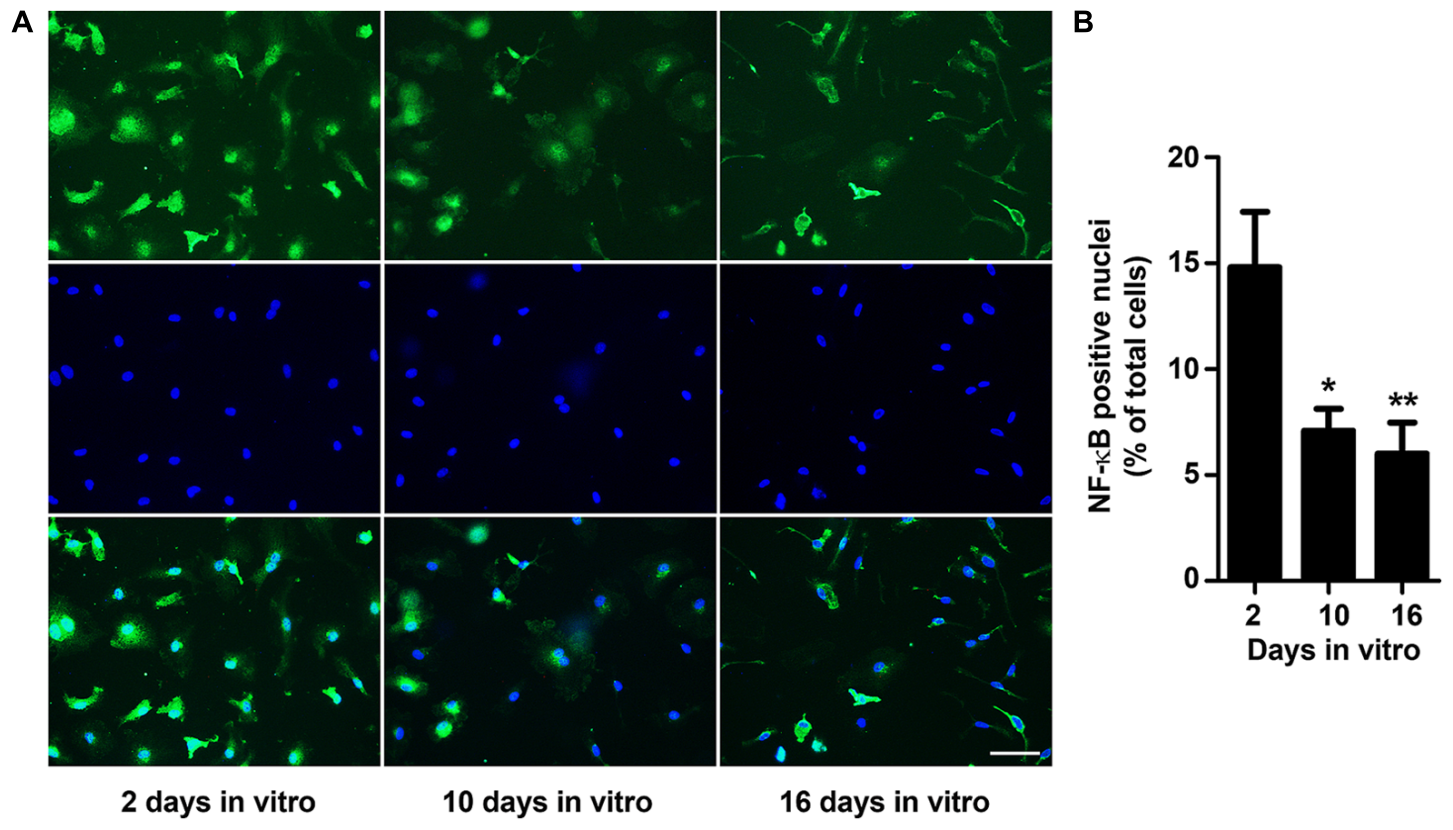
**NF-κB activation decreases with microglia aging in culture.** Microglial cells were kept in culture for 2, 10, and 16 days *in vitro* (DIV), immunostained for nuclear factor kappa-B (NF-κB; green) and their nuclei stained with Hoechst dye (blue). **(A)** Representative images at 2, 10, and 16 DIV. **(B)** Cells bearing a NF-κB-positive nuclei were counted and results expressed in graph bars as mean ± SEM. Cultures, *n* = 4 per group. *Post hoc* Bonferroni test, *p < 0.05 and **p < 0.01 vs. 2 DIV cells. Scale bar equals 50 μm.

Microglia play key immune-related duties, intervening through the production of anti-inflammatory compounds and trophic factors, by phagocytosing non-functional cells and debris, but also by releasing pro-inflammatory cytokines, depending on the stimuli. Production of several cytokines during microglial activation process is associated with the activation of the inducible transcription factor NF-κB (O’Neill and Kaltschmidt, 1997). To explore whether microglia morphological changes along the time in culture could be related with the cell activation state, we investigated NF-κB transactivation at the time points used to assess morphological alterations. Following microglia immunollabeling for p65 NF-κB subunit, we determined the number of NF-κB-positive nuclei as an indicator of its activation (**Figure 2**). Our results show that microglia express maximal NF-κB activation at 2 DIV decreasing significantly thereafter and reaching minimal levels at 16 DIV (~0.4-fold vs. 2 DIV, *p* < 0.01). These results corroborate the previous data in cell morphology and reinforce that microglia are highly reactive at 2 DIV but reduce their activation profile to a minimum state at 16 DIV. Thus, to settle that microglia at these *in vitro* stages may be associated to activated (2 DIV) and to age-like irresponsive cells (16 DIV), we additionally explored several markers that have been linked with age-related alterations in the dynamic behavior of microglia.

### AGED MICROGLIA SHOW A RESIDUAL MIGRATION ABILITY

**FIGURE 3:**
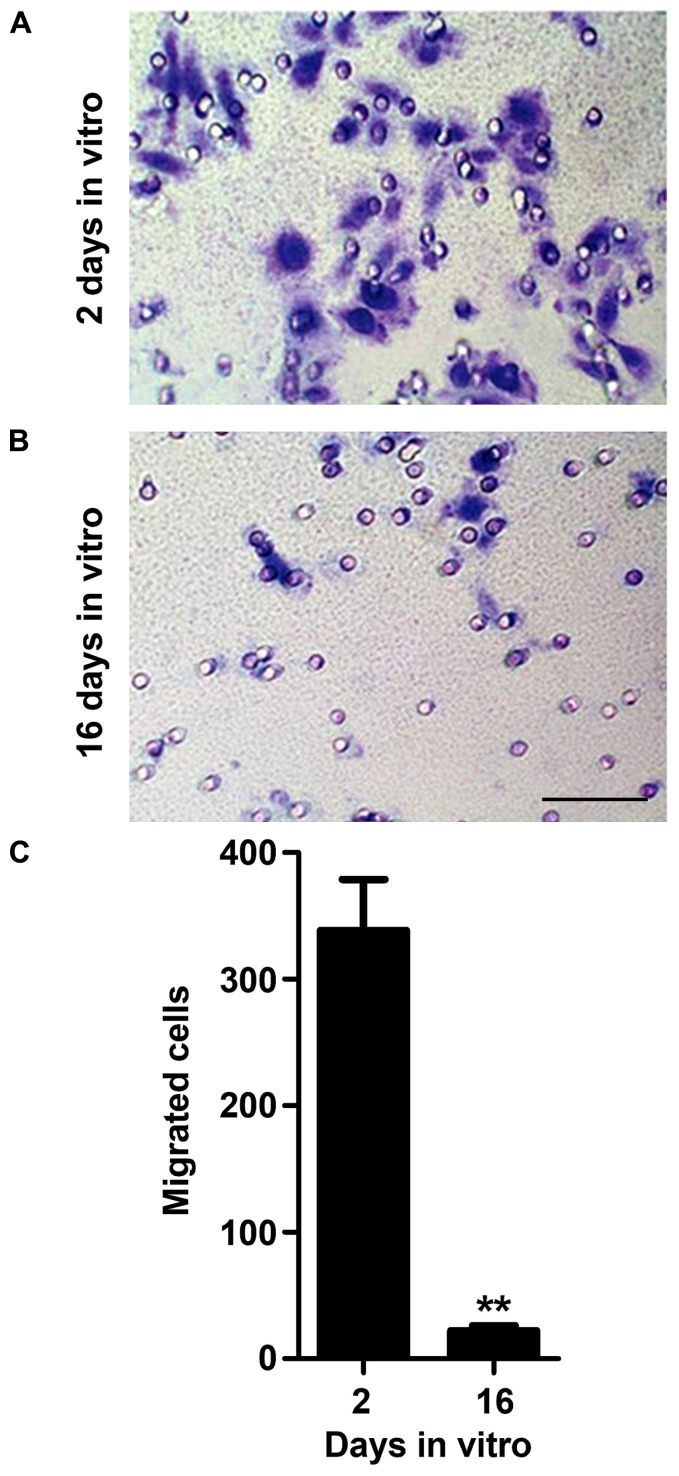
**Microglia migration ability decreases with cell aging in culture.** Microglial cells were kept in culture for 2 and 16 days *in vitro* (DIV) and then cellular chemotactic migration to 10 μM ATP was evaluated using the Boyden chamber method. Representative images of 2 **(A)** and 16 **(B)** DIV microglia that migrated towards ATP were visualized by Giemsa staining. Number of migrated cells was counted and results expressed in graph bars as mean ± SEM **(C)**. Cultures, *n* = 4 per group. *t*-test, **p < 0.01 vs. 2 DIV cells. Scale bar equals 50 μm.

Microglia directed migration towards regions of injury, also known as chemotaxis, is a property that seems to be more related to the classically (M1) and alternatively activated microglia (M2a; Lively and Schlichter, 2013). The release of chemotactic molecules upon brain damage, such as ATP, was indicated to participate in the recruitment of microglia toward lesion sites (Miller and Stella, 2009; Kettenmann et al., 2011). Nevertheless, it was reported that microglia respond to ATP regardless of their activation state (Lively and Schlichter, 2013). Hence, we evaluated the ability of 2 and 16 DIV microglia to migrate towards 10 μM ATP. As shown in **Figure 3**, 16 DIV microglia revealed a poor ability to migrate to ATP when compared to 2 DIV cells (~0.1-fold, *p* < 0.01). This finding points to a 2 DIV population of reactive microglia with capacity to migrate to local brain injury in contrast to the aged cells that lose invasion capacity property.

### AGED MICROGLIA SHOW REDUCED PHAGOCYTIC ABILITY

**FIGURE 4:**
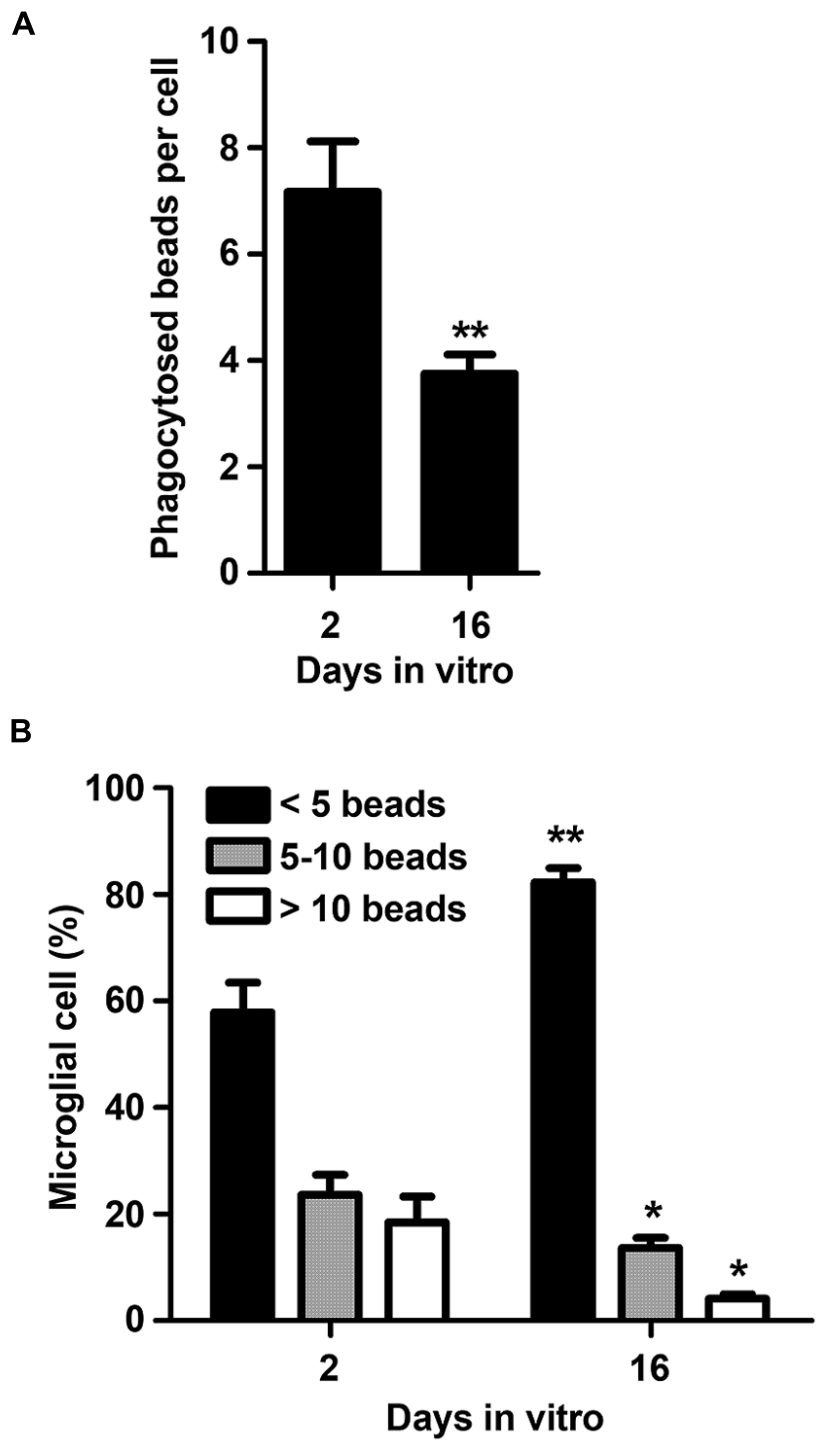
**Microglia phagocytic ability decreases with cell aging in culture.** Microglial cells were kept in culture for 2 and 16 days *in vitro* and then exposed to fluorescent beads to measure their phagocytic capacity. Number of phagocytized beads per cell **(A)** and the number of microglia phagocytosing <5, 5–10, and >10 beads **(B)** was counted and results expressed in graph bars as mean ± SEM. Cultures, *n* = 4 per group. *t*-test and *post hoc* Bonferroni test, *p < 0.05 and **p < 0.01 vs. 2 DIV cells.

Microglia are considered the professional phagocytes of the CNS, a function that is crucial along brain development, as well as in pathology and regeneration (Kettenmann et al., 2011). Therefore, and based on the previous results, we hypothesized that aging in culture could also have adverse effects on microglia phagocytic properties. As expected, 16 DIV microglia showed reduced engulfment ability when compared to 2 DIV cells (**Figure 4A**). Indeed, the average number of beads phagocytosed by each microglial cell was markedly reduced from 2 to 16 DIV (~0.5-fold, *p* < 0.01). In addition, we observed that aged microglia function less effectively than the 2 DIV cells based on the increased number of cells that engulf a small number of beads (*p* < 0.01) together with a decreased ability to digest 5 or more beads (*p* < 0.05; **Figure 4B**). Altogether these data suggest that *in vitro* aging of microglia obtained from neonatal mice change their dynamic behavior to a more inert or irresponsive phenotype compatible with an irresponsive/senescent cell.

### MICROGLIA RETAIN VIABILITY DURING *IN VITRO* AGING

Given our previous results we wondered whether the loss of microglia function by *in vitro* aging was a consequence of reduced cell viability. Therefore, we evaluated microglia cell death by flow cytometry following staining with annexin V-PE and 7-AAD, to differentiate the total amount of cells (adherent plus detached) into viable, early apoptotic and late apoptotic/necrotic cells. As shown in **Table 1**, we did not observe differences in cell death between the 2 and the 16 DIV microglia, confirming that changes in aged microglia response are not due to reduced viability but rather derive from a switch in cellular phenotype and in its properties.

**Table 1:** Viability of culturing microglia.

	Viable cells	Early-apoptotic cells	Late-apoptotic/necrotic cells
2 DIV	81.8 (±2.6)	9.7 (±0.3)	6.2 (±2.2)
16 DIV	81.7 (±3.0)	11.4 (±1.9)	7.4 (±1.6)

*All results are means ± SEM from at least four independent experiments. Microglial were kept in culture for 2 and 16 DIV. The percentage of viable microglia and microglia in early- or late-apoptosis/necrosis was determined by flow cytometry with phycoerythrin-conjugated annexin V (annexin V-PE) and 7-amino-actinomycin D (7-AAD). The three populations were distinguished as follows: viable cells (annexin V-PE and 7-AAD negative), early apoptotic cells (annexin V-PE positive and 7-AAD negative), and cells in late stages of apoptosis or dead cells (annexin V-PE and 7-AAD positive).*

### SUPPLEMENTARY FEATURES OF MICROGLIA REACTIVE ABILITY ARE REDUCED IN AGED CELLS

**FIGURE 5:**
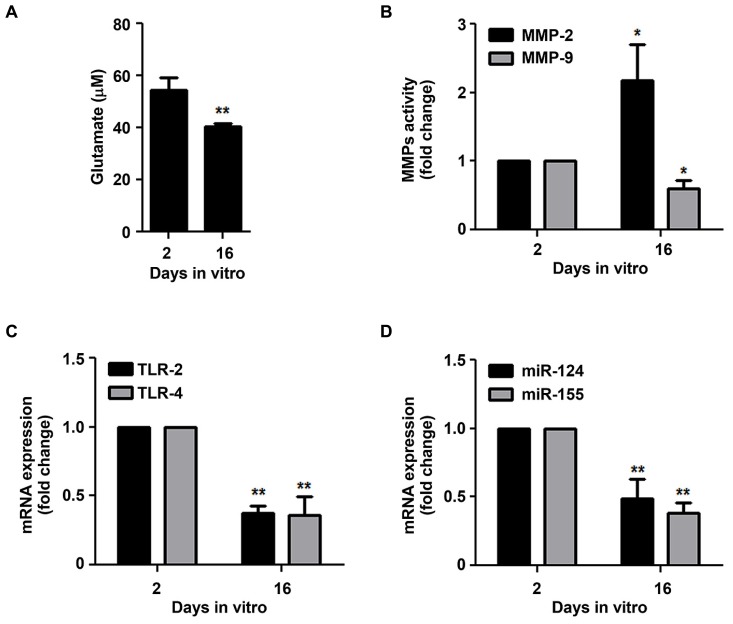
**Microglia supplementary features of reactive ability are reduced in aged cells.** Microglial cells were kept in culture for 2 and 16 days *in vitro* (DIV). **(A)** Extracellular glutamate levels were determined using a commercial kit. **(B)** Matrix metalloproteinases (MMP)-2 and MMP-9 activities were assessed by gelatin zymography. Expression of Toll-like receptor (TLR)-2 and TLR-4 **(C)**, as well as of microRNA (miR)-124 and miR-155 **(D)** was evaluated by Real-Time PCR. Results are expressed in graph bars as mean ± SEM. Cultures, *n* = 4 per group. *t*-test, *p < 0.05 and **p < 0.01 vs. 2 DIV cells.

Since 16 DIV microglia have shown decreased ability to respond to chemotactic signals and to phagocytose extracellular particles, features that were not related with loss of cell survival (**Table 1**), we next decided to evaluate whether microglia aged in culture would also present additional markers of reduced reactive ability. Glutamate was shown to be released by activated microglia (Noda et al., 1999; Barger et al., 2007; Takaki et al., 2012), reason why we decided to evaluate the extracellular content of glutamate. As depicted in **Figure 5A**, 16 DIV microglia showed to release lower levels of glutamate to the culture media than the 2 DIV cells (~0.7-fold, *p* < 0.01). Interestingly, when evaluating MMP-2 and MMP-9 activation in the extracellular media we verified that the influence of aging was also notorious (**Figure 5B**). Indeed we observed a marked increase of MMP-2 (~2.2-fold, *p* < 0.05) and a decrease of MMP-9 (~0.6-fold, *p* < 0.05) in the aged microglia when compared to 2 DIV cells. Again, the expression of TLR-2 and TLR-4 that is associated with microglia activation (Banks and Robinson, 2010; Liu et al., 2012) very much decreased in the 16 DIV microglia (~0.4-fold, *p* < 0.01, **Figure 5C**). Recently, immune regulation by miR-124 was indicated to downregulate microglia activation (Ponomarev et al., 2011) in contrast with miR-155 that was shown to have a pro-inflammatory role in microglia (Cardoso et al., 2012), to be related with the M1 phenotype (Ponomarev et al., 2013) and to be up-regulated upon activation (Lu et al., 2011). Corroborating previous findings, the decreased expression of both miR-124 and miR-155 in 16 DIV microglia as compared to 2 DIV cells (~0.5- and 0.4-fold, respectively, *p* < 0.01, **Figure 5D**) further reinforce that the cells become irresponsive/senescent when maintained in culture.

### 16 DIV MICROGLIA SHOW COMMON MARKERS OF SENESCENCE

**FIGURE 6:**
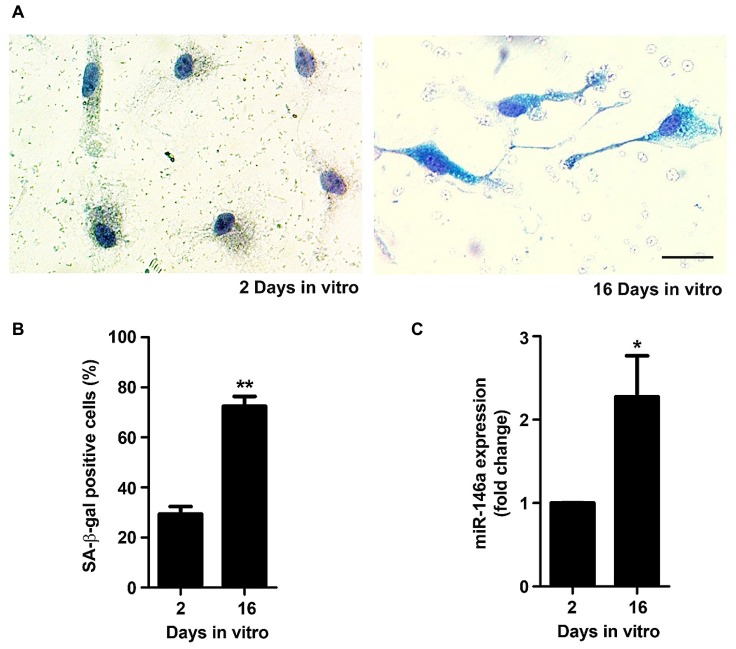
**Microglia aged in culture display signs of senescence, including increased senescent-associated β-galactosidase (SA-β-gal) activity and microRNA (miR)-146a expression.** Microglial cells were kept in culture for 2 and 16 days *in vitro* (DIV). Activity of SA-β-gal was determined using a commercial kit. **(A)** Representative images of 2 and 16 DIV microglia showing SA-β-gal staining. **(B)** SA-β-gal-positive cells were counted and results expressed in graph bars as mean ± SEM. **(C)** miR-146a expression was evaluated by Real-Time PCR. Results are expressed in graph bars as mean ± SEM. Cultures, *n* = 4 per group. *t*-test, *p < 0.05 and **p < 0.01 vs. 2 DIV cells. Scale bar equals 20 μm.

**FIGURE 7:**
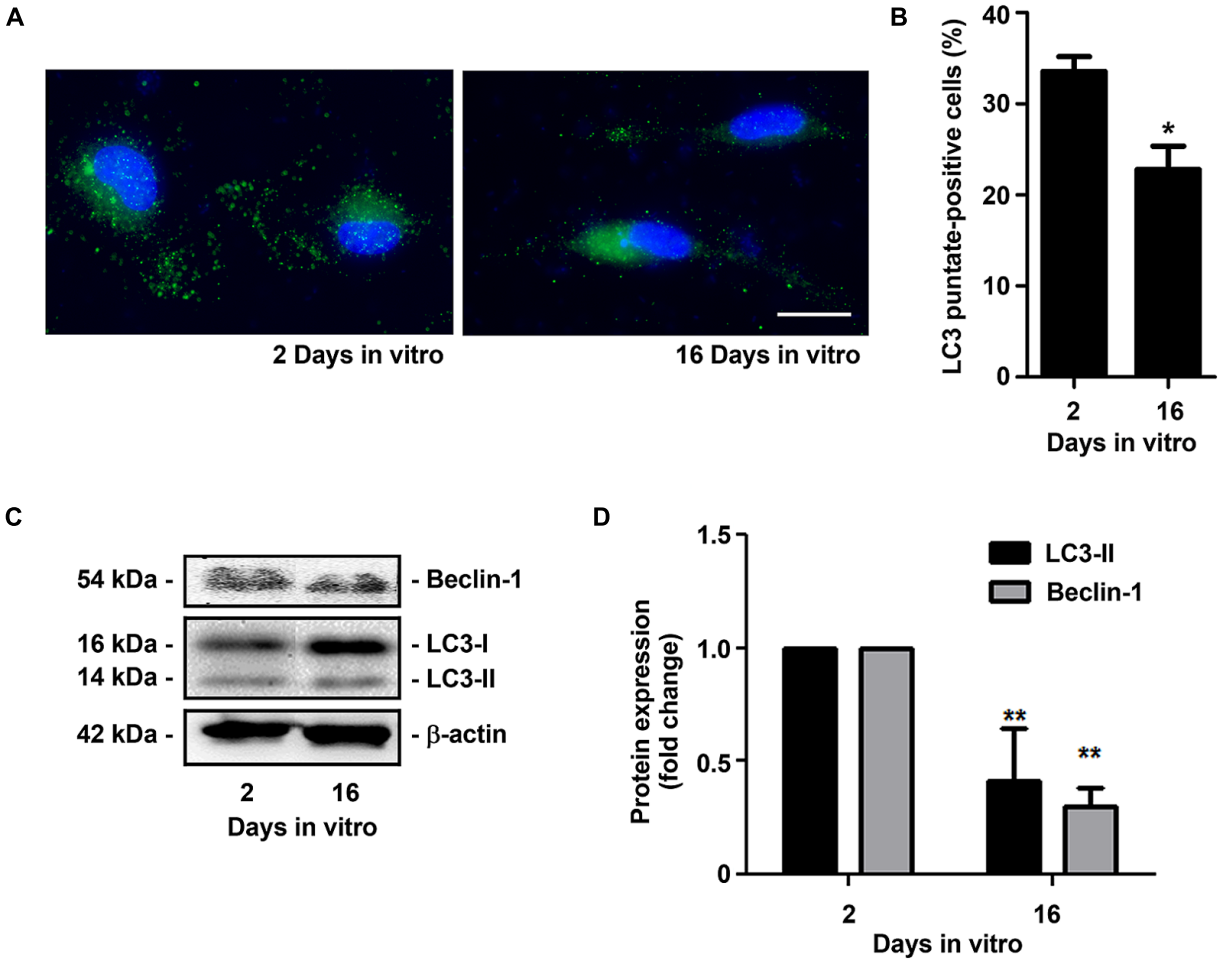
**Microglia aged in culture show reduced autophagic capacity.** Microglial cells were kept in culture for 2 and 16 days *in vitro* (DIV), immunostained for LC3 and total cell lysates were analyzed for the presence of LC3-II and Beclin-1. **(A)** Representative images of 2 and 16 DIV microglia showing LC3 punctates. **(B)** Cells displaying LC3 punctates were counted and results expressed in graph bars as mean ± SEM. **(C)** LC3-II/LC3-I and Beclin-1 protein expression was assessed by Western Blot and **(D)** densitometric data analysis is represented in graph bars as mean ± SEM. Cultures, *n* = 4 per group. *t*-test, *p < 0.05 and **p < 0.01 vs. 2 DIV cells. Scale bar equals 20 μm.

Senescent microglia have been described to become dysfunctional and less efficient in their neuroprotective effects during aging in the human brain and in AD (Streit and Xue, 2012; Krabbe et al., 2013). The main proposal of the present study was to obtain an experimental model able to reproduce irresponsive/senescent microglia that could be used for exploring detrimental effects by aging and associated-neurodegenerative diseases. As so, we decided to evaluate if the *in vitro* aged microglia displayed typical signs of cell senescence. The senescence phenotype has been associated with changes in cellular morphology, increased activity for SA-β-gal, permanent DNA damage, chromosomal instability and altered inflammatory secretome (Sikora et al., 2011). More recently, new biomarkers of age-associated senescence have been reported, including an increased expression of miR-146a in aged macrophages (Jiang et al., 2012) and a reduced capacity to undergo autophagy (Ma et al., 2011). Quantitative assay of SA-β-gal activity revealed that the percentage of positively stained cells markedly increased from 2 to 16 DIV (~2.5-fold, *p* < 0.01), as evidenced in **Figures 6A,B**. Similarly, we noticed a significant elevation in the expression of miR-146a along the cell aging in culture (~2.3-fold, *p* < 0.05, **Figure 6C**). Finally, we evaluated autophagic capacity by LC3 immunostaining. As it may be observed in **Figure 7A**, 2 DIV cells displayed an increased amount of LC3 punctates when compared to 16 DIV microglia. Counting of LC3 punctate-positive cells confirmed that a reduced number of 16 DIV cells were undergoing autophagy (~0.7-fold, *p* < 0.05, **Figure 7B**). Next, we evaluated the expression of LC3-II that is formed through lipidation of LC3-I during autophagy (Kabeya et al., 2000) and we additionally determined the Beclin-1 protein, recognized to have a central role in such process (Kang et al., 2011), by Western Blot (**Figures 7C,D**). Our results clearly show that LC3-II and Beclin-1 are markedly reduced in 16 DIV microglia when compared to 2 DIV cells (~0.4- and ~0.3-fold, respectively, *p* < 0.01), confirming a reduced autophagy by the aged microglia.

Overall, our data indicate that primary microglia harvested from neonatal mouse pups first evidence an increased reactive ability changing to an irresponsive/senescent cell when maintained in culture. Aged cells evidence a reduced ability to become activated, to migrate and to phagocytose, in parallel with markers of cellular senescence. Therefore, this *in vitro* model can be very useful in the exploitation of microglia reactivity and irresponsiveness to stimuli, respectively. In addition, changes in microglia miRNA signature may constitute a precious help in evaluating the key role of microglia as a determinant in age-associated CNS disorders and in modulating microglia dynamic properties.

## DISCUSSION

Experiments in this study were carried out to investigate age-specific differences in the dynamic functional profiles of neonatal microglia aged in culture, from 2 DIV up to 16 DIV. Here we show that microglia isolated from neonatal pups evidence markers of reactive ability at early time culture changing their phenotype along *in vitro* culture to less responsive cells that present senescence biomarkers and miRNA profiling characteristic of microglia deactivation. Collectively, our results indicate that microglia aging can be reproduced *in vitro* using long-term murine cultures, which may be used as a model to evaluate microglia performance in age-associated disorders, inasmuch due to the similar characteristics such mice cells evidence to human microglia (Torres-Platas et al., 2014).

Mouse primary neonatal microglial cultures have the advantage to more closely represent their in situ counterparts when compared to immortalized cells, although by growing in isolation lack the normal CNS microenvironment (Ni and Aschner, 2010). Indeed, primary cultured microglia are not oncogene immortalized and are differentiated in mixed glial cultures before isolation. The protocol here described originates microglial cultures that exceeds 97% purity and has been used as a model for activated CNS-resident microglia (Carson et al., 1998; Schmid et al., 2009) and to prepare polarized M1 and M2 phenotypes (Jang et al., 2013). Indeed, it was previously suggested that the isolation process is a sufficient stimulus to induce microglia activation (Cristóvão et al., 2010). There is a high controversy on whether neonatal microglia are less (Moussaud and Draheim, 2010) or more reactive than adult (Christensen et al., 2014) and aged cells (Njie et al., 2012). Discrepancies also exist in the scientific community based on studies that consider microglia overactivation and increased release of pro-inflammatory cytokines with age and neurodegenerative diseases (for review see Wong, 2013; Mosher and Wyss-Coray, 2014), in contrast with others evidencing dystrophic microglia and senescence (Streit et al., 2004; Streit and Xue, 2013), decreased phagocytosis (Floden and Combs, 2011; Li, 2013), lower reactivity to stimulation (Damani et al., 2011; Njie et al., 2012), delayed response to exogenous ATP and decreased microglial process motility (Hefendehl et al., 2014). Such contradictory results may be caused by different experimental sets and conditions. Moreover, most of the data were derived from experimental models using LPS-induced microglia activation, when it is well known that only a small amount is able to enter the brain parenchyma (Banks and Robinson, 2010). Therefore, the effects of peripheral immunostimulation by intravenously administered LPS dose are indirect and some of them mediated by the cells that comprise the BBB. Another important aspect to consider is that NSAIDs were only successful when administered before the development of neurodegeneration (Weggen et al., 2001). When administered in later stages of disease they showed to be detrimental (Martin et al., 2008). These findings may underlie a first proinflammatory stage in neurodegenerative diseases associated to neuroinflammmation, later switching to dysfunctional neurodegeneration associated with a loss of microglia dynamic properties. Indeed, neither the typical inflammatory nor the anti-inflammatory phenotypes were identified at end-stage amyotrophic lateral sclerosis (Nikodemova et al., 2013) and microglial dystrophy associated with their senescence (Flanary, 2005), as well as to aged and AD brain (Lopes et al., 2008).

Lack of knowledge on the molecular mechanisms implicated in aged microglia dysfunction and how it is related to an increased individual vulnerability to neurodegenerative diseases has hindered the development of effective therapy for preventing or even halting the CNS network degenerative process. Major problems to investigate such mechanisms are determined by the current *in vitro* microglial models using cell lines, primary microglia isolated from neonatal murine animals and *ex vivo* isolation from adult and aged brain. First models are not suitable for the research of neurodegenerative diseases where aging is crucial since long-term culture experiments are critical, and the last one only provides specific microglia subsets that resist to the isolation procedure (Moussaud and Draheim, 2010; von Bernhardi et al., 2011) or that are separated based on immunomagnetic cell sorting steps (Cardona et al., 2006). However, mixed microglial populations may coexist in the CNS and were also shown to be developed in culture (Szabo and Gulya, 2013; Gertig and Hanisch, 2014). In addition, microglia functionality from adult and aged animals is not well preserved, the yield is low and the cells undergo extensive cell death resulting in activation of the surviving population (von Bernhardi et al., 2011). The *in vitro* model we developed to obtain microglia senescence in primary culture has been likely used to identify aging-associated changes in fibroblasts at the molecular level (Chen et al., 2009). Finally, we have not used microglia culturing with astrocytes to avoid the complex interactions between these cells (Tanaka et al., 1999) that would be a disadvantage to assess natural microglia maturity and senescence. Therefore, establishment of well-defined stable *in vitro* cultures freshly isolated from neonatal mice and characterization of microglial phenotype with the time in culture may provide advantages over the other methods to determine aging microglial dynamics modifications and therapeutic approaches to recover microglial functionality.

Microglia morphology changed along *in vitro* maintenance from an almost exclusive round amoeboid shape to distinct polarized populations, including an increased number of ramified cells. In accordance, mixed primary glial cultures from embryonic rats have previously showed the existence of cells with an amoeboid morphology in the early stages of *in vitro* differentiation, which changed to mixed populations of amoeboid and ramified cell morphologies in older cultures (Szabo and Gulya, 2013). Interestingly, data from microglia isolated from different age animals also corroborate such findings with adult microglia presenting a more ramified morphology, in contrast with an amoeboid shape of embryonic and neonatal microglia (Lai et al., 2013). This is in line with *in vivo* data indicating that invading neonatal microglia have a predominant rounded morphology that differentiate with time into a surveying phenotype characterized by a small soma and highly branched processes (Hanisch and Kettenmann, 2007). Our aged microglia cultures besides exhibiting ramified and amoeboid cells also presented cells with a bipolar shape and shorter large processes. Morphological signs of microglia senescence with aging were observed *in vivo* and defined as abnormal morphological features, such as shortened, gnarled, beaded, or fragmented cytoplasmic processes, and loss of fine ramifications and formation of spheroidal swellings (Streit et al., 2004). Therefore, we hypothesize that such cells with shortened processes represent microglia with less ability to become reactive and should include a relevant population of senescent microglia.

The morphological changes of *in vitro* aging microglia occurred in parallel with a decrease in the transactivation of NF-κB. It is well known that this transcription factor is found throughout the cytoplasm, translocating to the nucleus upon activation triggering the transcription of target genes, such as the pro-inflammatory cytokines (O’Neill and Kaltschmidt, 1997). Therefore, maximal activation of NF-κB 2 days after isolation is consistent with an inflammatory phenotype that shifts to a deactivated microglia along with the time in culture. Intriguingly, although we showed a decreased NF-κB activation at 16 DIV, the activation of this transcription factor has been associated with the aging process. A recent report has shown that hypothalamic microglial NF-κB activation promoting a residual inflammatory status is required for systemic aging (Zhang et al., 2013). Nevertheless, a marked down-regulation of NF-κB was also observed in cultured senescent human WI-38 fibroblasts (Helenius et al., 1996). Considering that activators of the NF-κB signaling pathway are determinants of inflammation and aging process (Balistreri et al., 2013) and that CNS inflammation is present in the early stages of age-related disorders such as AD but disappears with disease progression (Streit et al., 2009), our *in vitro* aged microglia may represent a dystrophic and irresponsive phenotype whose functions have progressively declined as recently observed in mice with AD-like pathology (Krabbe et al., 2013).

The reduced migration observed for 16 DIV cells is in line with recent data showing that aged microglia become less dynamic with slower acute responses and lower rates of process motility (Damani et al., 2011). Here, we measured ATP-induced microglial chemotaxis, which occurs via P2X4R and P2Y12R purinergic receptors (Ohsawa et al., 2007). Interestingly, even considering that the expression of P2X4R in microglia is not age-dependent, the P2Y12R expression varies with animal age increasing to a maximum at 6–8 months and decreasing thereafter to extremely low levels at 13–15 months (Lai et al., 2013). Thus, it is possible that our aged microglia present reduced expression of purinergic receptors which may be in the origin of the reduced ability to migrate to ATP. Moreover, since it was demonstrated that monocyte chemoattractant protein-1 (MCP-1) produced downstream NF-κB activation is involved in the migration of microglia (Deng et al., 2009), based on the age-dependent reduction of NF-κB nuclear translocation we have observed it is reasonable to consider that the MCP-1-dependent migration pathway may also be affected.

Phagocytosis is crucial to maintain tissue homeostasis and innate immune balance, by ingesting both foreign pathogens and autologous apoptotic cells (Napoli and Neumann, 2009). Infectious pathogens are phagocytosed through TLRs or complement receptors to elicit the release of pro-inflammatory cytokines (Napoli and Neumann, 2009), while apoptotic cells or cellular debris are internalized through phosphatidylserine receptors, integrins or TREM2 to trigger immunosuppressive signaling with the release of anti-inflammatory cytokines (Li, 2012). During aging, clearance of both foreign pathogens and autologous apoptotic cells is diminished and has been associated with immunosenescence (Li, 2013). In accordance, microglia from aged mice also internalized less amyloid-β peptide (Aβ) than microglia from neonatal or young mice (Njie et al., 2012), corroborating our findings that 16 DIV microglia have a reduced ability to phagocytose possibly due to the manifestation of a senescent phenotype. Interestingly, microglial cells maintained in mixed primary neuronal-glial co-cultures were shown to phagocytose more when amoeboid than in the ramified form, a property that decreased during culturing (Szabo and Gulya, 2013). In agreement, we observed a shift to a more ramified phenotype with cell aging, which paralleled a reduced phagocytic ability.

Activated microglia were shown to release increased levels of glutamate (Noda et al., 1999; Barger et al., 2007; Takaki et al., 2012). However, several studies have shown lower glutamate concentration in older subjects when compared to younger individuals (Kaiser et al., 2005; Sailasuta et al., 2008; Chang et al., 2009) and an age-dependent decline of glutamate release in mice (Minkeviciene et al., 2008). This finding is in line with the reduced glutamate levels we obtained in aged cell cultures. Similarly, the increased activation of MMP-2 we observed in 16 DIV microglia was identified in senescent cells (Liu and Hornsby, 2007; Lu et al., 2009; Malaquin et al., 2013). In what concerns MMP-9 there is some discrepancy between authors. Some indicate increased activity with age (Simpson et al., 2013) and others a decrease (Bonnema et al., 2007; Paczek et al., 2008), as we obtained. Furthermore, we think that the marked reduced expression we obtained at 16 DIV microglia for TLR-2 and TLR-4 (0.5- and 0.4-fold, respectively), as compared to 2 DIV cells, define with no doubt that 16 DIV microglia will be less able to respond to LPS immunostimulation. Actually, TLR-4 that is critical for the recognition of LPS, as well as TLR-2 that also recognizes some LPS species, are inducers of microglia activation leading to the production of proinflammatory cytokines (Banks and Robinson, 2010; Liu et al., 2012). Curiously, the TLR-4 downregulation-mediated supression of TNF-α and IL-1β expression revealed to also be accompanied by the suppression of NF-κB (Yao et al., 2013).

MicroRNAs are an abundant class of highly evolutionarily conserved small non-coding RNAs that are involved in posttranscriptional gene silencing, regulating diverse biological processes (Ambros, 2004). miR-146a was first associated with the innate immune response as a negative feedback regulator in TLR signaling (Taganov et al., 2006), and more recently implicated in age-related dysfunction of macrophages (Jiang et al., 2012). Our results clearly showed that aged microglia express increased levels of miR-146a, thus corroborating their senescent phenotype. Interestingly, expression of miR-146a that has been associated with several neurodegenerative disorders (Sinha et al., 2011; Jiang et al., 2013), was found elevated in the aged mouse (Jiang et al., 2012; Olivieri et al., 2013), in the cerebrospinal fluid of AD patients (Alexandrov et al., 2012), and to be induced in microglia upon Aβ and inflammatory challenge (Li et al., 2011). As so, our *in vitro* old microglia reproduce the aging-associated phenotype encountered in late-life common disorders. Moreover, decreased miR-124 and miR-155 that revealed a negative correlation with age (Fichtlscherer et al., 2010; Noren Hooten et al., 2010; Smith-Vikos and Slack, 2012), parallelled by the enhanced miR-146a expression, further reinforce that 16 DIV microglia mainly represent aged-like microglia. In addition the reduced miR-124 obtained in these cells, indicated as being associated to the M2a-alternatively activated state (Freilich et al., 2013) and to inhibit inflammation (Prinz and Priller, 2014), strengthen their dormant/senescent phenotype. In contrast, the predominant amoeboid morphology together with increased NF-κB activation, cell migration, phagocytosis and the higher levels of miR-155 expression in 2 DIV microglia, as compared with aged cells, are indicative of a major representation of cells with a stressed/reactive phenotype. Indeed, a strong up-regulation of miR-155 expression was shown to have a pro-inflammatory role in microglia (Cardoso et al., 2012) and to drive the M1 phenotype (Ponomarev et al., 2013) corroborating the stressful properties of 2 DIV cultured cells.

Nowadays, changed morphology and increased activity of SA-β-gal of permanently growth arrested cells are considered cellular senescence markers (Sikora et al., 2011). In accordance, 16 DIV microglia displayed a marked increase of SA-β-gal activity when compared to 2 DIV cells. The activity of SA-β-gal was also associated with senescence-unrelated settings, such as contact inhibition and serum starvation (Severino et al., 2000). Nevertheless, as observed by the Iba-1 pictures, our microglia culture did not reach confluence and was not cultured under serum starvation, attesting that the increase of SA-β-gal activity results from a senescent phenotype. Indeed, decreased microglia migration, phagocytic ability, NF-κB activation and increased SA-β-gal, as we here observed, have been indicated as hallmarks of microglial aging and cell senescence (Mosher and Wyss-Coray, 2014).

Several neurodegenerative diseases are characterized by the formation of intracellular protein aggregates in affected brain regions, indicating a failure of protein degradation system (McCray and Taylor, 2008). Autophagy is a stress-induced catabolic process responsible for the degradation of long-lived proteins and damaged organelles (Levine and Klionsky, 2004) that was shown to decline with aging (Bergamini, 2006) and to determine cell and individual lifespan (Juhasz et al., 2007). A study using the senescence accelerated mouse prone eight, a rodent model of aging and senile dementia, showed a reduced autophagic activity by aging with long-lasting autophagosomes and increased LC3 expression (Ma et al., 2011). In accordance, affected neurons with abnormal autophagosomes (Lee, 2009) and impaired autophagy (Komatsu et al., 2006) were seen in neurodegeneration. We showed that 16 DIV microglia display a reduced amount of LC3 punctates suggestive of a decreased formation of autophagosomes. This finding was further corroborated by the decrease we also observed in the expression of Beclin-1 in the aged cells. Beclin-1 is known to intervene from autophagosome formation to autophagosome/endosome maturation but to also have other additional functions (Kang et al., 2011). Interestingly, Beclin-1 was recently considered to be required for efficient phagocytosis and to be reduced in microglia isolated from AD brains (Lucin et al., 2013), thus accounting to explain the reduced phagocytic ability in our 16 DIV cells and to such impairment in mice with AD-like pathology (Krabbe et al., 2013).

It is worth mentioning that the 2 and 16 DIV microglia differently react to some tested neurotoxins, as we antecipated. We used unconjugated bilirubin that has previously shown to induce the release of the pro-inflammtory cytokines TNF-α and Interleukin (IL)-1β from astrocytes and microglia in concentrations similar to those induced by 10 ng/ml LPS (Fernandes et al., 2004; Gordo et al., 2006; Brites et al., 2009), and Aβ at 50 nM, a concentration that was indicated to trigger microglia activation (Maezawa et al., 2011). The test was first directed to the expression of the high-mobility group box protein-1 (HMGB1) a mediator of inflammation directly correlated with NK-κB protein activation (Rovina et al., 2013). Both stimuli enhanced cellular HMGB1 expression in 2 DIV microglia (80 and 100% increase for bilirubin and Aβ, respectively; results not shown), whithout affecting the 16 DIV cells. Next, and similarly to what we have obtained for HMGB1, up-regulation of mRNA levels of IL-18 expression capable of more potently induce inflammatory response than IL-1β (Alboni et al., 2010) was again associated with the young/reactive microglia treated with bilirubin (60% increase over control) or Aβ (>100% increase over control) (data not shown), but no alterations were noticed in the aged cells.

Overall, we demonstrate that microglia isolated from neonatal mice and kept *in vitro* in long-term cultures switch from an activated/reactive phenotype to cells presenting aging-like alterations. Our results show that *in vitro* aged microglia change their morphology to a more ramified shape, with a reduced basal NF-κB activation, impaired migration and phagocytic abilities, low TLR-2 and TLR-4 expression, as well as reduced MMP-9 and glutamate efflux. This study is the first to provide the inflamma-miRNA signature for microglia aging in primary cultures. The cells evidenced decreased expression of miR-155 and miR-124, reduced autophagic capacity, and increased miR-146a expression and SA-β-gal activity, consistent with the existence of senescent cells at 16 DIV in culture. In conclusion, given the phenotypical changes observed for young/reactive and irresponsive/senescent microglia along the time in culture, the *in vitro* model of microglia aging could be of interest to assess how different signals may diversely modify cell functionality in separate microglia populations and to link increased age with risk for neurodegenerative diseases and other age-related phenomena.

## Conflict of Interest Statement

The authors declare that the research was conducted in the absence of any commercial or financial relationships that could be construed as a potential conflict of interest.
